# Reducing human error in global healthcare: leadership, learning, and system resilience

**DOI:** 10.1093/eurpub/ckag053

**Published:** 2026-03-19

**Authors:** Ayza Altaf, Enemona Jacob

**Affiliations:** School of Life and Medical Sciences, University of Hertfordshire, Hatfield, Hertfordshire, United Kingdom; Centre of Postgraduate Medicine and Public Health, University of Hertfordshire, Hatfield, Hertfordshire, United Kingdom

## Abstract

Human error in healthcare and public health remains a major contributor to patient harm and inefficiency globally. In high-income countries, it is estimated that 1 in 10 patients are harmed during hospital care, with nearly half of these incidents being preventable. In low- and middle-income countries (LMICs), the situation is even more concerning. The World Health Organization (WHO) has identified adverse events in healthcare as a major source of preventable harm in LMICs, highlighting persistent systemic weaknesses in quality of care and patient safety. While human errors occur, most patient harm stems from complex systemic factors rather than negligence. High-reliability organizations show that proactive leadership, transparent communication, and organizational learning reduce error likelihood and impact. Continuous quality improvement and simulation-based training strengthen resilience, enabling systems to anticipate and adapt to challenges. Technology-assisted tools, such as electronic health records and decision-support systems, further enhance error detection, though their success depends on integration and engagement. Building health system resilience therefore requires coordinated strategies that prioritize leadership, organizational learning, and adaptive design over punitive responses.

## Introduction

Human error remains a major contributor to adverse patient outcomes and inefficiency in healthcare systems globally. In high-income countries, ∼1 in 10 patients suffers harm during hospital care, with nearly half of these incidents considered preventable [1, 2]. In low- and middle-income countries (LMICs), these challenges are magnified by resource constraints, limited workforce capacity, and infrastructural deficiencies. The World Health Organization estimates that annually, LMICs experience ∼134 million adverse events in healthcare settings, resulting in 2.6 million deaths [3, 4]. These figures underscore the critical need to address unsafe medical practices, diagnostic errors, and lapses in treatment delivery as part of a systemic approach to patient safety.

While human errors are unavoidable, research indicates that most errors stem from complex systemic factors rather than personal negligence [5, 6]. Organizational structures, communication failures, and workflow inefficiencies interact to create environments in which errors occur. High-reliability organizations (HROs) demonstrate that proactive leadership, transparent communication, and organizational learning can mitigate these risks and reduce error impact [7–9]. By fostering adaptive and learning-focused cultures, healthcare systems can transform errors into opportunities for systemic improvement.

Despite increasing recognition of human error as a systemic issue, much of the existing literature remains fragmented, frequently addressing leadership, learning, or technology in isolation rather than as interrelated elements of health system resilience. This paper addresses the issue of preventable harm in the international health sector by examining how organizational learning, leadership practices, and resilient system design collectively impact the prevention and remediation of human error. This issue is specifically critical in LMICs, where system-level vulnerabilities and capacity constraints intensify the consequences of system failures. In this paper, the term ‘leadership’ is discussed broadly to encompass both organizational and clinical leadership, with particular emphasis on transformational and distributed leadership approaches. This paper aims to contribute by consolidating evidence across organizational learning, leadership and resilience frameworks to showcase a cohesive, systems-oriented perspective on human error. In doing so, the article enhances understanding of how healthcare organizations can move beyond reactive, blame-oriented approaches toward proactive strategies that reinforce safety, sustainability and adaptability across diverse global contexts.

In this paper, **human error** is conceptualized as unintentional actions or decision-making processes that contribute to unsafe care, emerging mainly from interactions between individual and technological, organizational and environmental system conditions rather than sole individual negligence [5, 10]. **Leadership** is understood as the clinical and organizational capacity to influence safety culture, decision-making, and learning processes with specific emphasis on distributed and transformational leadership approaches encouraging staff engagement, accountability, and psychological safety [8, 11, 12]. **Health system resilience** can be defined as the capacity of healthcare systems to absorb, anticipate and adapt to, and recover from disruptions while sustaining safe and effective service delivery [13, 14].

Consistent with resilience engineering, this paper adopts a Safety-II perspective, defining safety as the healthcare system’s ability to maintain effective performance under variable and constrained conditions, rather than merely preventing adverse events [15].

## Understanding human error as a systemic phenomenon

Traditional approaches to human error often focus on individual accountability. However, contemporary research supports a systems perspective [5, 10]. Complex interactions among personnel, technology, processes, and environmental factors contribute to adverse events. The Swiss Cheese Model illustrates how latent failures align to create opportunities for errors, emphasizing the importance of robust system design [5, 16]. Addressing human error therefore requires interventions targeting both individual competencies and health system resilience. Embedding safety-focused practices into organizational processes ensures that errors are detected early and mitigated before reaching the patient [17, 18].

## Leadership and safety culture

Leadership is central to fostering a culture of safety and health system resilience. Transformational leadership, characterized by vision, motivation, and empathy, is associated with improved patient outcomes and enhanced staff engagement [8, 11]. Leaders influence safety not solely through policy enforcement but by modelling behaviours, encouraging reporting, and promoting accountability. Distributed leadership further enhances resilience by empowering frontline staff to take ownership of safety initiatives and contribute to decision-making processes [12].

Effective leadership is especially critical in LMIC contexts, where resource constraints and systemic pressures may exacerbate errors. By cultivating psychological safety and trust, leaders ensure that errors are openly discussed and addressed without fear of punitive repercussions [8, 19]. In doing so, leadership transforms error management from a reactive process into a proactive learning opportunity.

## Organizational learning and resilience

Continuous organizational learning is integral to building health system resilience. Structured approaches, such as continuous quality improvement (CQI) and Plan–Do–Study–Act (PDSA) cycles, enable organizations to systematically identify hazards, implement interventions, and evaluate outcomes [20, 21]. Simulation-based training, particularly in operating theatres and other high-risk environments, allows healthcare professionals to rehearse complex scenarios, refine decision-making, and strengthen teamwork skills [20, 22].

Consistent with the Safety-II approach, errors are identified as unavoidable but manageable to avoid adverse outcomes if systems are designed to detect and respond effectively [13, 23]. In LMICs, promoting resilience involves targeted workforce development, standardized protocols, and adaptive resource allocation to mitigate the impact of systemic constraints [3, 24, 25].

## Technology-enhanced error prevention

Technological tools offer significant potential to reduce human error when appropriately integrated into clinical workflows. Electronic health records (EHRs) and machine-learning-based decision support systems can detect errors in medication administration, flag contraindications, and support diagnostic accuracy [24, 26]. Studies demonstrate that predictive models using EHR data improve early detection of patient safety events, enabling timely intervention [24, 27].

However, technology alone is insufficient. Systems must be designed to align with human capabilities, workflows, and organizational culture. Poorly implemented tools, alert fatigue, and insufficient training may paradoxically introduce new errors [28]. Leaders must therefore ensure that digital tools are user-centred, effectively embedded, and accompanied by training to maximize their safety potential [13, 29].

## Challenges and strategic approaches in LMICs

LMIC healthcare systems face amplified risks due to limited resources, infrastructure gaps, and high patient-to-staff ratios [3, 5, 30]. Strategies to mitigate human error in these contexts include strengthening infrastructure, providing ongoing staff training, implementing standardized protocols, and fostering a safety-oriented organizational culture [3, 8, 31]. High-impact, low-cost interventions, such as surgical and medication checklists, double-verification processes, and structured reporting systems, have proven effective in reducing preventable harm [5, 20, 32].

International collaboration, knowledge sharing, and adoption of high reliability organization (HRO) principles can support LMICs in cultivating resilient and safe healthcare systems. Context-specific adaptations are essential to address local challenges while maintaining alignment with global best practice standards [33, 34].

## Building health system resilience

Health system resilience encompasses structural, procedural, and cultural dimensions. Redundancy, flexible staffing, and continuous monitoring allow systems to anticipate and respond to errors effectively [8, 20, 35]. As discussed earlier, leadership and learning are intertwined with resilience; effective leadership creates environments in which staff feel empowered to act, while continuous organizational learning mechanisms ensure that improvements are institutionalized [8, 20, 13].

Balancing standardization with adaptability is essential. Overly rigid protocols may stifle innovation, whereas flexible procedures allow frontline teams to improvise safely under complex or unexpected conditions [36]. When aligned with organizational learning, resilience engineering approaches combined with predictive analytics, simulation, and CQI ensure that both anticipated and unanticipated errors are managed proactively [24, 13, 37].

## Conclusion

Reducing human error in global healthcare requires a systemic, multi-dimensional approach. Leadership, learning, and resilience are interdependent pillars that underpin safe, high-performing healthcare systems. As demonstrated in this paper, integrating leadership that supports psychological safety with resilient system design and continuous learning processes enables healthcare systems to adapt to complexity and eradicate preventable harm.

This integrated approach is highly critical in LMICs where resource constraints and systemic vulnerabilities heighten the consequences of failure. Fostering organizational learning, strengthening leadership capacity and aligning technological and structural aspects within a resilient framework have important implications for health policy and practice, promoting more sustainable, equitable, and safe care across diverse global contexts.

## Data Availability

No new data were generated or analysed in this article.
